# Incidental findings on prostate MRI in a population-based screening setting

**DOI:** 10.1186/s13244-025-02147-7

**Published:** 2025-11-24

**Authors:** Victoria Huang, Hang Dang, Erik Thimansson, Thomas Jiborn, Fredrik Jäderling, Anna Lantz, Rebecka Arnsrud Godtman, Jonas Wallström, Ola Bratt

**Affiliations:** 1 Department of Urology, Institute of Clinical Science, Sahlgrenska Academy, University of Gothenburg, Gothenburg, Sweden; 2 Diagnostic Radiology, Department of Translational Medicine, Lund University, Malmö, Sweden; 3 Department of Radiology, Helsingborg Hospital, Helsingborg, Sweden; 4 Department of Urology, Helsingborg Hospital, Helsingborg, Sweden; 5 Department of Molecular Medicine and Surgery (MMK), Karolinska Institutet, Solna, Sweden; 6 Department of Radiology, Capio St Görans Hospital, Stockholm, Sweden; 7 Department of Pelvic Cancer, Karolinska University Hospital, Stockholm, Sweden; 8 Department of Urology, Sahlgrenska University Hospital, Gothenburg, Sweden; 9 Department of Radiology, Sahlgrenska University Hospital, Gothenburg, Sweden; 10 Department of Radiology, Institute of Clinical Science, Sahlgrenska Academy, University of Gothenburg, Gothenburg, Sweden

**Keywords:** Prostate, Prostate cancer, Screening, Magnetic resonance imaging, Incidental findings

## Abstract

**Objective:**

To describe the frequency and types of incidental findings on prostate MRI in a screening setting.

**Materials and methods:**

Prostate MRI reports from 2020 to 2024 for men aged 50–56 years were collected from three regional organised prostate cancer testing (OPT) programmes in Sweden. Incidental findings were categorised as suspicious for extra-prostatic malignancy, otherwise likely clinically relevant or of low or no clinical relevance that do not motivate contact with the screened man. Confidence intervals (CI) were calculated for proportions of scans with an incidental finding. Chi-square testing was used to test inter-regional differences of reported findings.

**Results:**

At least one incidental finding was described in 119/1202 (9.9%) MRI reports. Ten reports described two incidental findings. Most (112/129, 87%) were categorised as of low or no clinical relevance, with inguinal hernia and colon diverticulosis being the most common. Proportions of these findings varied significantly (*p* = 0.005) between the regions: 47/355 (13%; 95% CI 10–17%), 47/539 (8.7%; 95% CI 6.5–11%) and 15/308 (5.8%; 95% CI 3.5–9.0%). Overall, 17/1202 (1.4%) of the reports described a suspected extra-prostatic malignancy or otherwise clearly clinically relevant incidental finding. Suspected extra-prostatic malignancy findings were four suspected tumours in the rectum, four suspected tumours in the bladder and two bone metastases with an unknown primary tumour.

**Conclusion:**

Screening prostate MRI in men in their fifties yields few incidental findings of clear clinical importance. Reporting of incidental findings of low/no clinical relevance varies between centres. Consensus-based guidelines are needed to define which types of incidental findings should be reported and notified to the screened individuals.

**Critical relevance statement:**

Screening prostate MRI detects few incidental findings of clear clinical relevance. Findings of low or no clinical relevance are variably reported, which calls for consensus-based guidelines on which types of incidental findings on screening prostate MRI should be reported.

**Key Points:**

- No previous study has reported incidental findings on prostate MRI in a population-based screening setting.
- Screening prostate MRI detects few incidental findings of clear clinical relevance.
- Incidental findings of low or no clinical relevance are variably reported across centres.
- There is a need for consensus-based guidelines for which types of incidental findings on screening prostate MRI should be reported and notified to the screened individual.

**Graphical Abstract:**

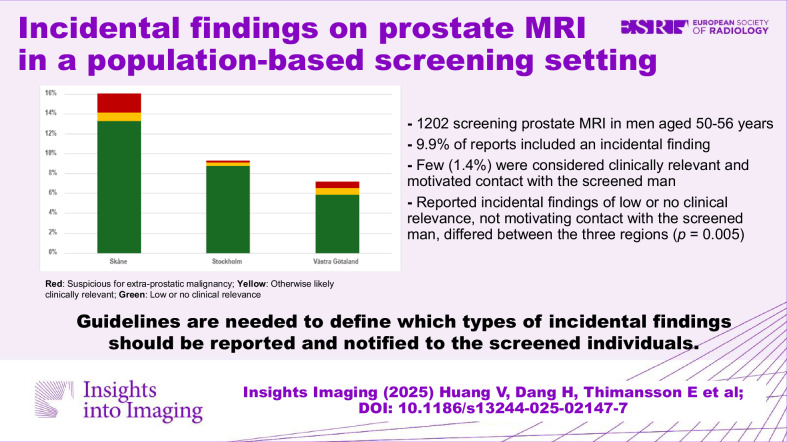

## Introduction

Screening for prostate cancer has been evaluated in randomised clinical trials, in which a systematic biopsy was used to investigate men with a raised prostate-specific antigen (PSA) value [1, 2]. This diagnostic pathway led to unacceptable overdiagnosis of small, well-differentiated tumours. Modern prostate cancer diagnostics include magnetic resonance imaging (MRI) of the prostate for men with a raised PSA to identify tumours for a targeted biopsy. Randomised screening trials with MRI in the diagnostic pathway are ongoing [3, 4], and the European Union in December 2022 recommended the member states to evaluate organised screening for prostate cancer with such a pathway [5]. In Sweden, regional, population-based, organised prostate cancer testing (OPT) programmes with an MRI-based diagnostic pathway were started in 2020 [6, 7].

The introduction of imaging in the diagnostic pathway for prostate cancer has led to incidental findings of various conditions, ranging from clearly clinically insignificant findings, such as compact bone islands, to highly significant findings such as invasive bladder cancer [8–13].

The management of incidental findings on imaging differs between the clinical setting and a screening setting. In screening, presumably healthy individuals are actively invited to a diagnostic evaluation for a specific condition, in this case, prostate cancer. This means that extra care must be taken when considering which types of incidental findings the screened individuals should be notified of.

With the increasing use of prostate MRI for screening purposes comes a need for guidelines for which incidental findings to report and which to communicate to the screened individual. A first step is to establish the frequency and types of reported incidental findings in a screening setting, but no report of this has yet been published. We therefore evaluated this in three Swedish regional Organised Prostate Cancer Testing (OPT) programmes, and whether reporting of incidental findings varies across centres.

## Materials and methods

The regional Swedish population-based OPT programmes have been described in detail elsewhere [7]. They are provided by the public healthcare providers, who actively invite entire birth cohorts by letter. Men in OPT with a PSA ≥ 3 ng/mL are referred for a prostate MRI (in Region Stockholm, an evaluation of the Stockholm3 test was carried out in 2024, which meant that some men with PSA ≥ 3 ng/mL did not have an MRI). The MRI scans are done and reported at the same radiology departments that do the routine clinical prostate MRI scans in public healthcare. The radiologists’ experience level varies; most are either sub-specialists or dedicated OPT readers, but some are general abdominal radiologists.

This non-interventional, retrospective study included reports from participants’ first prostate MRI in the OPT programmes in Sweden’s three most populated regions (Region Skåne, Region Västra Götaland and Region Stockholm) through the years 2020 to 2024. Most invited men were aged 50 years, but some were aged 52 years (reinvited 2 years after their first invitation) or 56 years (first invitation). Men undergoing MRI in OPT were identified in the national register SweOPT. We estimated that over 2000 scans were done in these years, but considered about 1000 reports to be sufficient and therefore randomly selected half of the scans from each region.

Region Skåne and Region Västra Götaland used a prostate-specific online template for reporting [6, 14]. These templates did during the study period include an item for incidental findings that included the options “suspected tumour in rectum or urinary bladder”, “rectal inflammation”, “sigmoid diverticulosis”, “inguinal hernia”, “history of inguinal repair with net” and “other findings”, but free text about incidental findings could be added to the report sent to the OPT office.

We retrieved the MRI reports and categorised the described incidental findings as suspicious for an extra-prostatic malignancy, otherwise likely clinically relevant and requiring notification to the screened individual, or of low or no clinical relevance and therefore not requiring notification to the screened individual. The categorisation was inspired by previous publications of incidental findings on routine clinical prostate MRI [8–13] and finalised after repeated discussions among the authors. One report mentioned “prostatitis” as an incidental finding, but this finding was excluded as all types of findings in the prostatic parenchyma should be categorised as findings according to the Prostate Imaging–Reporting and Data System v2.1 (PI-RADS) [15].

The routines for reporting clinically irrelevant incidental findings vary across radiology departments and healthcare regions. We calculated 95% confidence intervals (CIs) for the proportions of reports with an incidental finding by category. If two incidental findings were reported for one scan, the most severe finding was used for categorisation. The inter-regional difference of findings with no or low clinical relevance was analysed with the Chi-Square test.

The study was approved by the Swedish Ethical Review Authority (2024-01684-02).

## Results

A total of 1202 MRI reports were reviewed: 539 from Region Stockholm, 355 from Region Skåne and 308 from Region Västra Götaland. The age of the men was 50–56 years (median 50 years) at the time of the MRI. A total of 119/1202 men (9.9%; 95% CI 8.3–12%) had at least one incidental finding reported. Ten of these men had two incidental findings reported, which amounts to a total of 129 incidental findings. The proportion of men with a reported incidental finding of low or no clinical relevance only was 104/1202 (8.7%; 95% CI 7.1–10%). Of the total incidental findings, 112/129 (87%) were categorised as of low or no clinical relevance. Most of these were either an inguinal hernia 39/112 (35%) or colon diverticula 35/112 (31%). Other findings of low or no clinical relevance included various types of cysts 10/112 (8.9%) and various infrequent findings (Table 1).

**Table 1:** Incidental findings reported on prostate magnetic resonance imaging (MRI) in three regional organised prostate cancer testing (OPT) programmes in Sweden

Red = suspicious for an extra-prostatic malignancy. Yellow = otherwise likely clinically relevant, requiring notification to the man. Green = of low or no clinical relevance, not requiring notification to the man. Total number green; total number yellow; and total number red in bold values. Bold values = total numbers

**Fig. 1:**
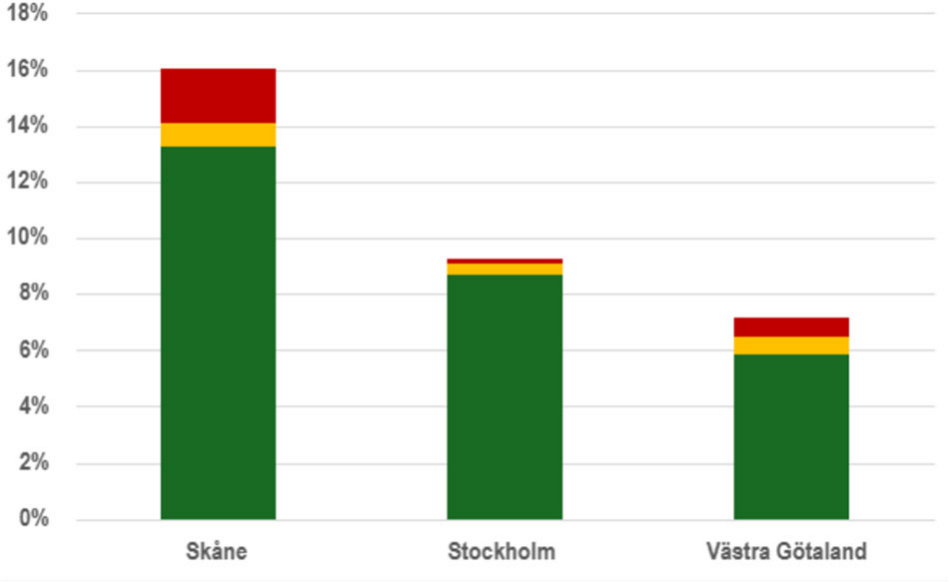
Incidental findings reported on prostate magnetic resonance imaging (MRI) in three regional organised prostate cancer testing (OPT) programmes in Sweden. Red = suspicious for an extra-prostatic malignancy; yellow = otherwise likely clinically relevant and requiring notification to the man; green = of low or no clinical relevance, not requiring notification to the man. The difference between regions in the proportion of incidental findings with low or no clinical relevance was statistically significant (*p* = 0.005)

The proportion of incidental findings of low or no clinical relevance significantly varied across regions (*p* = 0.005): Region Skåne 47/355 (13%; 95% CI 10–17%), Region Stockholm 47/539 (8.7%; 95% CI 6.5–11%) and Region Västra Götaland 18/308 (5.8%; 95% CI 3.5–9.0%) (Fig. 1).

**Fig. 2:**
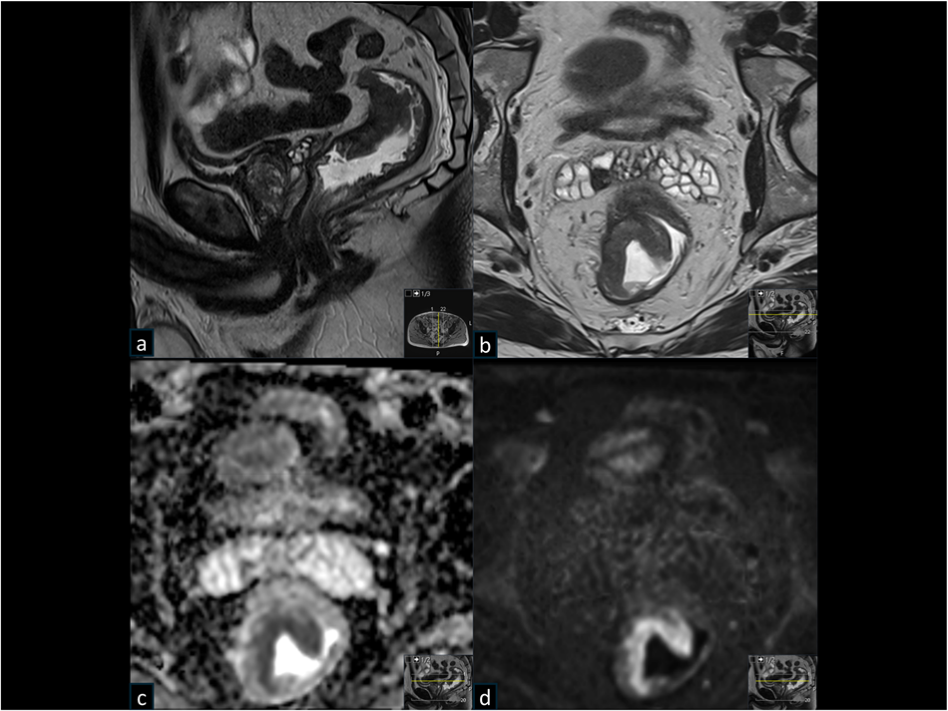
A 50-year-old man from OPT with PSA 4.00 µg/L. Incidental finding of semi-circumferential rectal tumour, which was operated (pT3bN0). T2W sag (**a**), T2W trans axial (**b**), ADC (**c**) and high b value DWI b1400 s/mm² (**d**)

Ten of the 129 (7.8%) reported incidental findings were suspicious for an extra-prostatic malignancy, and additional 7/129 (5.4%) as otherwise likely clinically relevant (Fig. 1). This means that 17/1202 (1.4%; 95% CI 0.8–2.2%) men had an incidental finding categorised as clinically relevant and requiring notification to the screened individual for subsequent further investigation or follow-up. The 10 findings suspicious for extra-prostatic malignancy were a suspected tumour in the rectum (*n* = 4) (Fig. 2), a suspected tumour in the urinary bladder (*n* = 4), and bone metastasis (*n* = 2) with an unknown primary tumour.

## Discussion

In this study from a population-based prostate cancer screening setting in Sweden, only 1.4% of men in their fifties had an incidental finding on prostate MRI that was clearly clinically relevant and led to some sort of intervention or follow-up investigation. The proportion of reported incidental findings of low or no clinical relevance, considered not requiring notification to the man, varied across regions from 5.8 to 13%. This variation reveals a need to establish guidelines for reporting and managing incidental findings on prostate MRI when introducing prostate cancer screening programmes.

The overall proportion of reported incidental findings was smaller than what has been published for prostate MRI in routine clinical practice [8–13]. One study reported that as many as 53% of clinical prostate MRI scans showed some kind of incidental finding [8], but in that study, the radiologists were reviewing the scans retrospectively to identify incidental findings. The proportion of incidental findings categorised as clinically relevant was also smaller in our study than in other studies (1.4% of all scans); others have reported 3–7% [8–13]. In one study, only 1 out of 249 men (0.4%) younger than 65 years had a clearly clinically relevant incidental finding, but a further 14% had a potentially relevant one [8]. One reason for the smaller proportion in our study is that the men were younger than in the other studies [8–13], as both clinically relevant and irrelevant incidental findings increase with age [8, 10, 11]. Another reason is probably that the MRI scans in our study were done in a screening setting, where reporting of incidental findings is usually considered not necessary or, indeed, not justified [16]. In a clinical setting, men who have a prostate MRI may initially have contacted healthcare because of pelvic symptoms such as pain or urinary urgency. Many of the conditions that we considered clinically irrelevant in a screening setting, for example, hernias, may cause such symptoms and could therefore be relevant in a clinical setting.

Possible causes of the inter-regional differences in the proportion of reported low or non-clinically relevant findings in our study include variations in regional or local routines, varying use of structured reporting templates, and individual radiologist preferences. One likely cause is that many of the radiologists reading MRI in the Region Västra Götaland OPT programme have long experience of reading prostate MRI for men in their fifties in the Gothenburg-2 screening trial (4), where reporting of incidental findings is restricted. These radiologists have shared their experiences with other radiologists in the region who read OPT MRI. Another possible cause is that in Region Skåne and Region Västra Götaland, but not in Region Stockholm, prostate MRI in OPT is reported using a structured reporting template with a multiple-choice function regarding incidental findings, including inguinal hernias and diverticulosis [14]. Discussions about incidental MRI findings in Swedish OPT have occurred, partly inspired by the present study. In 2025, it led to a revision of the MRI report template so that now only incidental findings that require clinical follow-up or an intervention are to be reported.

The watershed when categorising incidental findings in a screening setting is whether the screened individual should be notified about the finding or not. This decision has ethical and medicolegal consequences. In 2023, the Swedish national working group for OPT initiated a smaller multidisciplinary working group of radiologists, urologists, and a medical ethicist that was commissioned to write a guideline for how incidental MRI findings should be managed in OPT. This guideline was published in February 2025 as an annex (see Supplement) to the Swedish OPT recommendations, which are available in English online. The guideline advises that only clearly clinically relevant incidental findings should be reported on OPT MRI. It also advises that the invitation to OPT should include the following text: “Sometimes the examination shows something outside the prostate which may be of significance for your health. In this case we will contact you.” The main motives for these recommendations were (1) that the invited men had been specifically offered screening for prostate cancer, nothing else, (2) that investigations of incidental findings and may cause anxiety and physical harm, and (3) that the workload for the healthcare would be substantial if a great proportion of men in a full-scale screening programme were to have follow-up for various incidental findings without any proven health benefit.

Two incidental findings that required some discussion among the authors before they were categorised were a testicle in the inguinal canal and a case of polycystic kidney disease. Arguments for categorising them as not clinically relevant were that these conditions were most likely already known by the men, that the risk of malignancy in a non-descended testicle is small in men over 50 years of age, and that other available imaging reports showed that the polycystic disease was diagnosed and followed up. Nonetheless, both these conditions were categorised as likely clinically relevant with the motivation that although the risk of malignancy is small, it is not negligible, and that although the polycystic kidney disease was already diagnosed, it could in other cases be undiagnosed.

The strength of our study is mainly the large, population-based sample of reports from prostate MRI in a screening setting, and that it included regions in different parts of the country. A weakness is that we have no information about the results of further investigations of the reported incidental findings. Limitations include that only scans for men in their early or mid-fifties were assessed. The reason was that few older men are yet invited to OPT in Sweden. This means that the proportions with reported incidental findings in our study are not generalisable to older populations. We plan to evaluate older men when there are sufficient scans from older men. Furthermore, the reviewed MRI reports were all from the participant’s first MRI in OPT, so our results do not apply to follow-up MRI in a screening programme.

## Conclusion

Screening prostate MRI in men in their fifties yields few incidental findings of clear clinical importance. The proportion of men with an incidental finding of low or low clinical relevance varies across centres, which suggests that radiologists have different routines and thresholds for mentioning incidental findings in their reports. Our results call for consensus-based guidelines for the reporting and management of incidental findings on screening prostate MRI, particularly for which incidental findings motivate notification to the screened individual for further investigation or follow-up.

## Supplementary information

ELECTRONIC SUPPLEMENTARY MATERIAL

## Data Availability

Electronic copies of the reviewed MRI reports are kept by one author in each of the three regions. As the reports include the personal identification number of the scanned individual, they are not available on request.

## Abbreviations

CI: Confidence interval
MRI: Magnetic resonance imaging
OPT: Organised prostate cancer testing
PI-RADS: Prostate Imaging–Reporting and Data System
