# Supplementary material for: Incidental findings on prostate MRI in a population-based screening setting
Source: Insights Imaging. 2025 Nov 24;16:261. doi: 10.1186/s13244-025-02147-7 (PMC12644368; doi:10.1186/s13244-025-02147-7)
Supplement: Supplementary file 1 — ELECTRONIC SUPPLEMENTARY MATERIAL [file 13244_2025_2147_MOESM1_ESM.pdf]

Incidental Findings on Prostate MRI in a Population-based Screening Setting

ELECTRONIC SUPPLEMENTARY MATERIAL

Supplementary table: Short description of the MRI protocols used in the three different regions

	Field strength	T2W Covering the prostate	DWI High b-value	DCE	T1W Covering the entire pelvis
Region Skåne	1.5 or 3T	Axial + at least one orthogonal plane	≥1400 s/mm2	Not performed	yes
Stockholm region	1.5 or 3T	Axial + sagittal	≥1400 s/mm2	Not performed	no
Västra Götaland region	1.5 or 3T	Axial + sagittal + coronal	≥1400 s/mm2	Not performed	yes
